# A Novel Approach to the Synthesis of 1,3,4-Thiadiazole-2-amine Derivatives

**DOI:** 10.3390/molecules26175159

**Published:** 2021-08-25

**Authors:** Tatiana S. Kokovina, Svyatoslav Y. Gadomsky, Alexei A. Terentiev, Nataliya A. Sanina

**Affiliations:** 1 Institute of Problems of Chemical Physics, The Russian Academy of Sciences, 142432 Chernogolovka, Russia; kota6711@yandex.ru (T.S.K.); alexei@icp.ac.ru (A.A.T.); sanina@icp.ac.ru (N.A.S.); 2 Faculty of Fundamental Physical and Chemical Engineering, Lomonosov Moscow State University, 119991 Moscow, Russia; 3 Medicinal Chemistry Science and Education Center, Moscow Region State University, 141014 Mytishchi, Russia

**Keywords:** 1,3,4-thiadiazole-2-amine, thiadiazoles, polyphosphate ester, cyclodehydration reaction, one-pot synthesis

## Abstract

The main purpose of the study was the development of a new method for synthesis of 1,3,4-thiadiazol-2-amine derivatives in a one-pot manner using the reaction between a thiosemicarbazide and carboxylic acid without toxic additives such as POCl_3_ or SOCl_2_. The reaction was investigated in the presence of polyphosphate ester (PPE). It was found that, in the presence of PPE, the reaction between the thiosemicarbazide and carboxylic acid proceeds in one-pot through three steps with the formation of corresponding 2-amino-1,3,4-thiadiazole. Using the developed approach five, 2-amino-1,3,4-thiadiazoles were synthesized. The structures of all compounds were proven by mass spectrometry, IR, and NMR spectroscopies.

## 1. Introduction

In recent years, there has been a noticeable increase in research interests in the synthesis and biological application of various derivatives of 2-amino-1,3,4-thiadiazoles. It has been shown that these compounds exhibit antitumor [1,2,3,4], antibacterial [1,5,6], antifungal [6], and antiparasitic [7,8] activities. In addition, there are several examples where 2-amino-1,3,4-thiadiazoles demonstrate inhibitory activity against carbonic anhydrase [9] and alpha-glycosidase [10], which is important for the treatment of glaucoma and diabetes mellitus type 2.

**Scheme 1:**
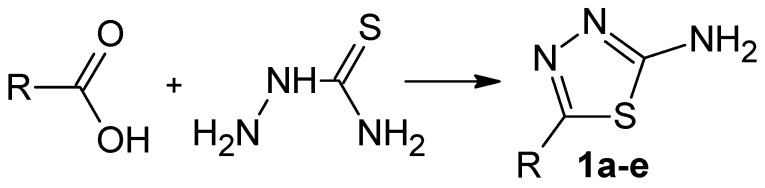
The reaction for 1,3,4-thiadiazol-2-amine derivatives synthesis.

A comprehensive list of approaches to synthesis of thiadiazoles is described in the review work [11]. There are several one-pot synthesis methods based on the reaction between thiosemicarbazides and carboxylic acids (Scheme 1): (i) reaction in ionic liquids [12]; (ii) reaction in excess of POCl_3_ [4]; (iii) reaction in polyphosphoric acid [13]; and (iv) reaction in a mixture of phosphorus pentoxide and methanesulfonic acid [14]. None of these one-pot synthesis methods are universal because of known limitations: (i) high production cost of ionic liquids [15]; (ii) high toxicity of POCl_3_ [16]; (iii) greater suitability of polyphosphoric acid for nitriles than for carboxylic acids [13]; and (iv) reactivity of methanesulfonic acid which can lead to byproduct formation [17,18]. Thus, the development of cost-efficient and safe one-pot methods for 2-amino-1,3,4-thiadiazoles synthesis remains relevant. It was shown recently that polyphosphate ester (PPE) is suitable for the synthesis of five-membered nitrogen-containing heterocyclic structures under mild conditions at a temperature not higher than 85 °C [19]. Based on the general concept of the reaction mechanism of PPE [19], one might expect that this reagent might also be used to carry out the reaction depicted in Scheme 1. In this regard, our work aimed at investigating the reaction between thiosemicarbazide and different carboxylic acids in the presence of PPE as a new approach to a 2-amino-1,3,4-thiadiazole one-pot synthesis.

## 2. Results

It was shown for the first time that the reaction of thiosemicarbazide with carboxylic acids in the presence of PPE proceeds with the formation of 2-amino-1,3,4-thiadiazoles in accordance with Scheme 1. We studied experimental conditions to find an optimal amount of PPE and dilution solvent. We found that it is necessary to use no less than 20 g of PPE for each 5 mmol of the carboxylic acid, otherwise the target thiadiazole does not form. We also found that chloroform is a convenient dilution solvent for the reaction since its insertion which gives a homogeneous reaction mixture and simplifies temperature control. The latter is very important since the temperature for the reactions using PPE must be below 85 °C [19]. Table 1 presents the results of the approach developed for 2-amino-1,3,4-thiadiazole synthesis based on Scheme 1. Table 1 shows that the synthesis conditions are applicable for both aromatic and aliphatic carboxylic acids. However, the poor solubility of some dicarboxylic acids in the reaction mixture can significantly complicate the process. For instance, it was found that using terephthalic acid is not suitable for the described approach, because most of the precursor (more than 70%) remains as an undissolved precipitate at the end of the experimental procedure.

## 3. Discussion

A possible mechanism of the reaction (Scheme 1) in the presence of PPE was proposed as a result of difficulties that arose during the experiment with 3-phenylpropionic acid. 

The ^1^H NMR spectrum of 1b clearly had signals of an impurity (see Figure S6a in supplementary materials). The specific integral intensity (per one proton) of the NH_2_ protons of the target compound was almost 30% lower than expected based on the aromatic proton signal, which might indicate that an impurity with a structure similar to 1b made a noticeable contribution to the aromatic signal. We hypothesized that the impurity was 2-(3-phenylpropanoyl) hydrazine-1-carbothioamide, i.e., the product of the thiosemicarbazide acylation by 3-phenylpropionic acid. It is known [20] that 2-(3-phenylpropanoyl)hydrazine-1-carbo -thioamide is an intermediate for 1b, and it can be converted into the thiadiazole by treatment in acidic medium. To verify this assumption, the product mixture 1b was treated with a hydrochloric acid solution. As a result, all signals of the impurity disappeared from the 1b ^1^H NMR spectrum (see Figure S6 in SI), which confirmed our hypothesis.

**Scheme 2:**
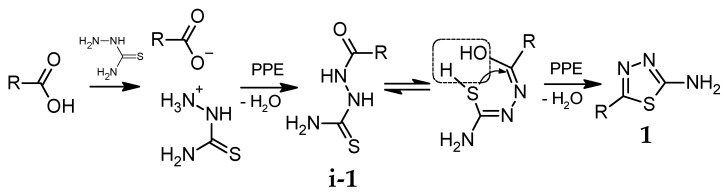
A possible pathway for the reaction of thiosemicarbazide with carboxylic acids in the presence of PPE.

To prove the 2-step nature of the discussed reaction, we synthesized 2-benzoylhydrazine-1-carbothioamide i-1a (intermediate for 1a) using PPE (the synthesis of the intermediate for 1a required adjusting the reaction conditions because the impurity is not observed under the optimized reaction conditions). We found the experimental conditions for the reaction between benzoic acid and thiosemicarbazide with predominant formation of i-1a. The reaction proceeds to the desired end point if PPE is added to a mixture of benzoic acid and thiosemicarbazide in chloroform, i.e., the salt of benzoic acid and thiosemicarbazide is formed prior to PPE addition. Taking these conditions into account, the following steps of the reaction between carboxylic acid and thiosemicarbazide in the presence of PPE can be assumed (Scheme 2): (1) salt formation; (2) dehydration of the formed salt to intermediate i-1; and (3) cyclodehydration of i-1 to thiadiazole 1.

## 4. Materials and Methods

IR spectra were acquired on a Bruker Alpha FT-IR spectrometer (all samples were analyzed directly without dilution in KBr). ^1^H NMR spectra were acquired on a Bruker DRX-500 in DMSO-*d_6_* with TMS as the internal standard. Mass spectra were recorded on a Finnigan MAT INCOS 50 mass spectrometer with direct sample injection (EI ionization, 70 eV). Polyphosphate ester (PPE) was synthesized according to the described method [21]. For IR spectra comparisons the SciFinder access to BIO-RAD data base was used [22].

Synthesis of compounds **1a–e** (General method). To a hot (60 °C) solution of 5-mmol carboxylic acid (2.5 mmol for dicarboxylic acid) in a mixture of polyphosphate ester (20 g), chloroform (30 mL) and 5 mmol of thiosemicarbazide was added. The reaction mixture was refluxed for 10 h, then 15 mL of distilled water was added to the mixture and the residual PPE was neutralized by NaHCO_3_. 

*5-Phenyl-1,3,4-thiadiazol-2-amine* (**1a**). The formed precipitate was filtered off, washed with chloroform and hexane. Yield 0.57 g (64.4%) of colorless crystals. Mass spectrum, *m/z* (*I*_rel_, %): 177 [M]+ (100), 121 (9.4), 104 (6.2), 77 (6.5), 74 (14.7), 28 (81.4). IR spectrum, *ν*, cm^−1^: 3256, 2966, 1633, 1508, 1467, 1379, 1333, 1263, 1138, 1057, 1001, 978, 913, 793, 758, 686, 623, 565, 496, 438, 383. ^1^H NMR (DMSO-*d_6_*), ppm (*J*, Hz): 7.37–7.51 (5H, m, NH_2_ and Ar-H); 7.77 (2H, d, *J* = 7.9, Ar-H).

*5-(2-Phenylethyl)-1,3,4-thiadiazol-2-amine* (**1b**). The organic layer was separated, followed by distillation of chloroform. The residue was refluxed in 5 mL of 10% hydrochloric acid for 5 h to purify from the traces of the intermediate, then the pH of the solution was adjusted to 8–9, and the resulting precipitate was filtered off. Yield 0.49 g (47.8%) of colorless crystals. Mass spectrum, *m/z* (*I*_rel_, %): 205 [M]+ (82,0), 206 (15.8), 204 (44.1), 188 (5.9), 130 (6.6), 128 (11.2), 127 (5.3), 115 (10.0), 114 (37.7), 104 (9.0), 103 (7.3), 99 (6.8), 92 (9.7), 91 (100), 89 (9.8), 78 (7.6), 77 (14.8), 74 (13.0), 65 (30.4), 63 (12.3), 62 (5.3), 60 (40.2), 59 (22.1), 58 (26.6), 51 (7.2), 42 (8.6), 39 (5.8), 28 (22.9), 27 (7.9). IR spectrum, *ν*, cm^−1^: 3675, 3270, 3110, 2970, 2901, 1627, 1517, 1496, 1452, 1393, 1336, 1212, 1158, 1048, 907, 743, 699, 666, 623, 526, 487, 433, 382. ^1^H NMR spectrum (DMSO-*d_6_*), ppm (*J*, Hz): 2.96 (2H, t, *J* = 7.6, CH_2_); 3.13 (2H, t, *J* = 7.6, CH_2_); 6.99 (2H, s, NH_2_); 7.21 (1H, t, *J* = 7.0, Ar-H); 7.24-7.33 (4H, m, Ar-H).

*5-(Phenoxymethyl)-1,3,4-thiadiazol-2-amine* (**1c**). The formed precipitate was filtered off and treated with 10% hydrochloric acid solution according to the procedure described for (**1b**). Yield 0.46 g (44.4%) of colorless crystals. Mass spectrum, *m/z* (*I*_rel_, %): 207 [M]+ (15,3), 114 (100), 94 (13.3), 77 (8.0), 65 (11.1), 60 (16.2), 59 (16.8), 58 (11.1), 39 (8.4), 28 (6.6). IR spectrum, *ν*, cm^−1^: 3662, 3248, 3099, 2972, 1634, 1589, 1525, 1506, 1493, 1455, 1363, 1301, 1245, 1222, 1171, 1139, 1076, 1041, 1002, 882, 842, 800, 750, 687, 672, 625, 530, 505, 417, 372. ^1^H NMR spectrum (DMSO-*d_6_*), ppm (*J*, Hz): 5.29 (1H, s, CH_2_); 7.00 (1H, t, *J* = 7.3, Ar-H); 7.05 (2H, d, *J* = 7.9, Ar-H); 7.28 (2H, s, NH_2_); 7.31 (2H, t, *J* = 7.9, Ar-H).

*5-[(Naphthalen-2-yl)methyl]-1,3,4-thiadiazol-2-amine* (**1d**). The formed precipitate was filtered off, washed with chloroform and hexane. Yield 0.081 g (67.2%) of colorless crystals. Mass spectrum, *m/z* (*I*_rel_, %): 241 [M]+ (100), 242 (15.5), 240 (86.7), 224 (12.8), 200 (18.2), 199 (92.4), 198 (42.3), 172 (16.7), 171 (26.2), 167 (30.7), 166 (71.6), 165 (20.4), 153 (16.1), 152 (48.9), 141 (61.9), 140 (21.3), 139 (143.5), 115 (71.6), 74 (25.3), 63 (12.3), 60 (30.9), 43 (20.7). IR spectrum, *ν*, cm^−1^: 3661, 2987, 2901, 1631, 1521, 1497, 1450, 1394, 1231, 1149, 1057, 871, 799, 790, 772, 738, 716, 668, 643, 567, 542, 516, 493, 470, 423, 410, 381, 373. ^1^H NMR (DMSO-*d_6_*), ppm (*J*, Hz): 4.63 (2H, s, CH_2_); 7.00 (2H, s, NH_2_); 7.46-7.57 (4H, m, Ar-H); 7.89 (1H, d, *J* = 7.3, Ar-H); 7.97 (1H, d, *J* = 7.5, Ar-H); 8.14 (1H, d, *J* = 7.9, Ar-H).

*5,5’-(Butane-1,4-diyl)di(1,3,4-thiadiazol-2-amine)* (**1e**). The formed precipitate was filtered off and treated with 10% hydrochloric acid solution, according to the procedure described for (1b). Yield 0.45 g (70.3%) of colorless crystals. Mass spectrum, *m/z* (*I*_rel_, %): 256 [M]+ (5.8), 181 (21.1), 142 (29.9), 141 (45.7), 130 (6.8), 129 (39.9), 128 (100), 116 (17.7), 115 (45.0), 114 (9.3), 99 (24.5), 85 (5.9), 75 (15.3), 74 (25.2), 72 (5.5), 71 (11.1), 60 (54.4), 59 (21.6), 58 (17.1), 47 (11.0), 45 (13.2), 43 (22.1), 42 (12.2), 41 (16.2), 39 (12.2), 32 (6.0), 29 (13.1), 28 (25.4), 27 (15.8). IR spectrum, *ν*, cm^−1^: 3265, 3081, 2973, 1634, 1521, 1495, 1455, 1408, 1341, 1233, 1183, 1056, 866, 834, 689, 619, 587, 443, 401. ^1^H NMR spectrum (DMSO-*d_6_*), ppm (*J*, Hz): 1.63-1.68 (4H, m, CH_2_); 2.79-2.83 (4H, m, CH_2_); 6.99 (4H, s, NH_2_).

*2-Benzoylhydrazine-1-carbothioamide* (**i-1a**). To a boiling mixture of benzoic acid (1 g, 8.2 mmol) and thiosemicarbazide (0.75 g, 8.2 mmol) in 6 mL of chloroform, 1 g of PPE was added. The resulting mixture was stirred at 85 °C for an hour (without backflow condenser). Then, the reaction mixture was diluted with 5 mL of distilled water, followed by pH neutralization with sodium bicarbonate. The resulting precipitate was filtered off, washed with hot chloroform (2 × 10 mL) and 10% methanol in chloroform (2 × 10 mL). Yield 0.65 g (40.24%) of colorless crystals. IR spectrum, *ν*, cm^−1^: 3415, 3137, 1700, 1683, 1637, 1602, 1580, 1546, 1497, 1468, 1249, 1174, 1085, 1069, 1025, 894, 817, 800, 703, 683, 603, 566, 490, 406, 392. ^1^H NMR spectrum (DMSO-*d_6_*), ppm (*J*, Hz): 7.49 (2H, t, *J* = 7.6, Ar-H); 7.58 (1H, t, *J* = 7.3, Ar-H); 7.64 (1H, s, C(S)-NH_2_); 7.87 (1H, s, C(S)-NH_2_); 7.92 (2H, d, *J* = 7.4, Ar-H) 9.35 (1H, s, -C(O)-NH-NH-C(S)-); 10.36 (1H, s, -C(O)-NH-NH-C(S)-).

## 5. Conclusions

A new method for the synthesis of 2-amino-1,3,4-thiadiazoles by the reaction of a carboxylic acid with thiosemicarbazide in the presence of PPE was developed. The reaction proceeds through the intermediate formation of the thiosemicarbazide acylation product with subsequent cyclodehydration to the target heterocycle. The experimental conditions for the reaction between benzoic acid and thiosemicarbazide in the presence of PPE with predominant formation of intermediate for the thiadiazole, 2-benzyl hydrazine-1-carbothioamide were found. The new approach has potential for extended application: (1) PPE can be utilized as a mild additive for thiosemicarbazide acylation reaction by carboxylic acids; and (2) the thiosemicarbazide acylation products used for synthesis of thiadiazoles can be also utilized for triazoles synthesis [23].

## Figures and Tables

**Table 1:** List of 2-amino-1,3,4-thiadiazoles synthesized using the developed approach (Scheme 1).

Precursor (RCOOH)	Thiadiazol 1	Yield, %
Benzoic acid	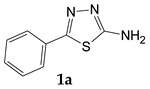	64.4
3-Phenylpropionic acid	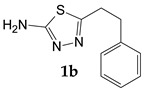	47.8
Phenoxyacetic acid	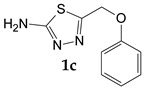	44.4
2-Naphthaleneacetic acid	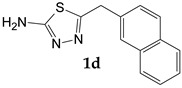	67.2
adipic acid	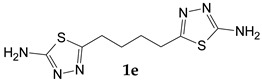	70.3

## Data Availability

The authors confirm that the data supporting the findings of this study are available within the article and its Supplementary Materials.

## Supplementary Materials

All the spectra (Figures S1–S17) are available online.

## Sample Availability

Samples of the compounds **1a**–**e** and **i**–**1a** are available from the authors.
