# Supplementary material for: A Novel Approach to the Synthesis of 1,3,4-Thiadiazole-2-amine Derivatives
Source: Molecules. 2021 Aug 25;26(17):5159. doi: 10.3390/molecules26175159 (PMC8434302; doi:10.3390/molecules26175159)
Supplement: Supplementary file 1 [file molecules-26-05159-s001.zip › molecules-1347402-supplementary.pdf]

Supplementary material

A novel approach to the synthesis of 1,3,4-thiadiazole-2-amine derivatives

Tatiana S. Kokovina [1,2], Svyatoslav Y. Gadomsky [1,*], Alexey A. Terentiev [1,2,3] and Nataliya A. Sanina [1,2,3]

1 Institute of Problems of Chemical Physics of the Russian Academy of Sciences, 142432 Chernogolovka, Moscow region, Russian Federation;
2 Lomonosov Moscow State University, 119991 Moscow, Russian Federation;
3 Medicinal Chemistry Science and Education Center of Moscow Region State University, 141014 Mytishchi, Russian Federation
* Correspondence: gadomsky@icp.ac.ru.

5-Phenyl-1,3,4-thiadiazol-2-amine (1a)

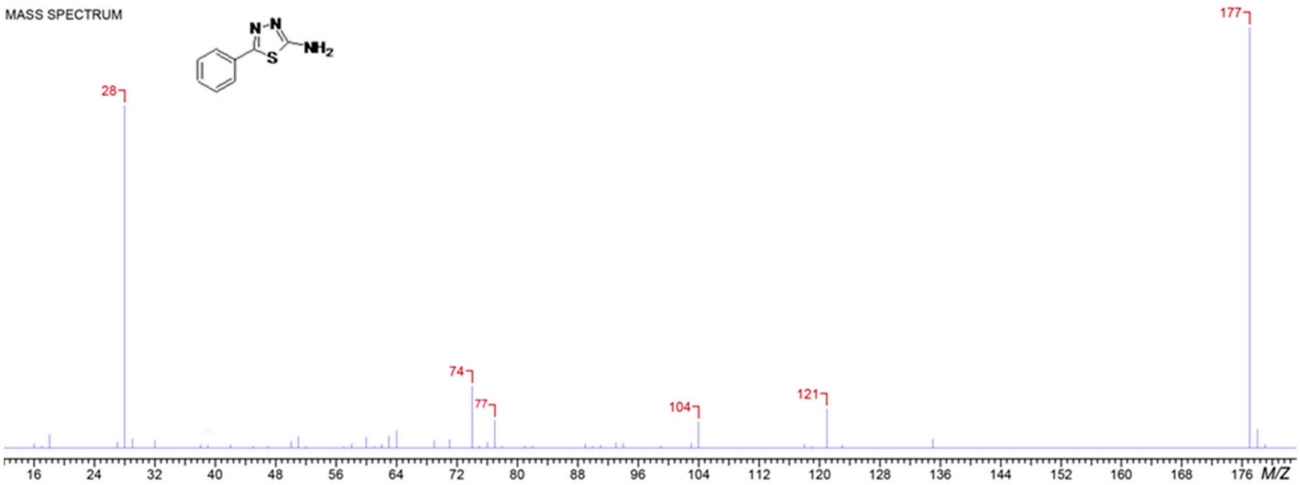

Figure S1. Mass spectrum of 5-phenyl-1,3,4-thiadiazol-2-amine (**1a**).

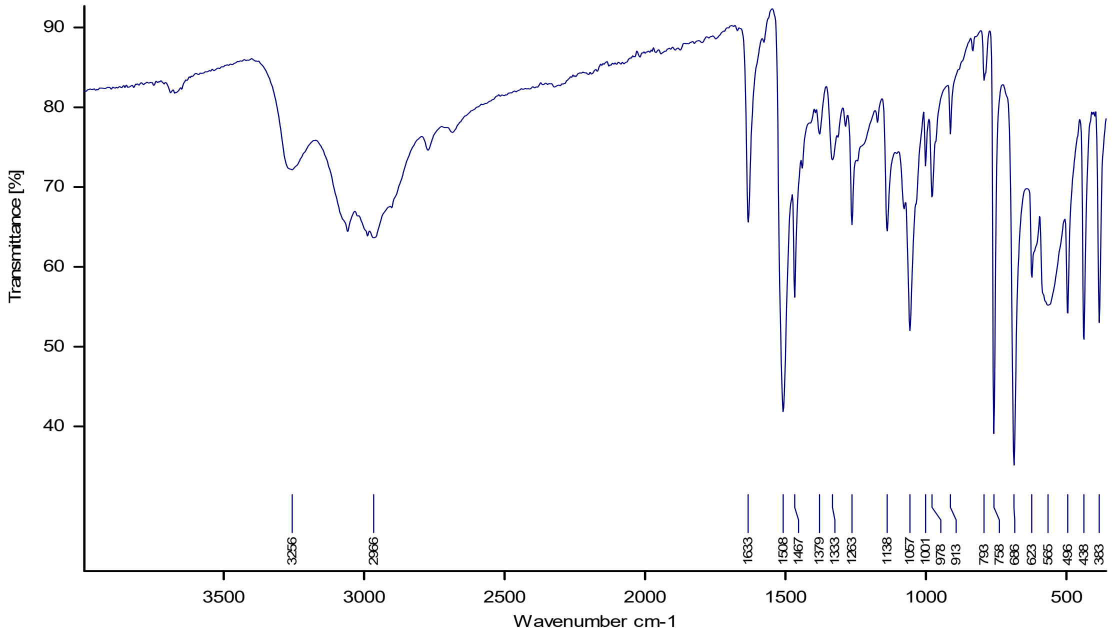

Figure S2. IR spectrum of 5-phenyl-1,3,4-thiadiazol-2-amine (**1a**).

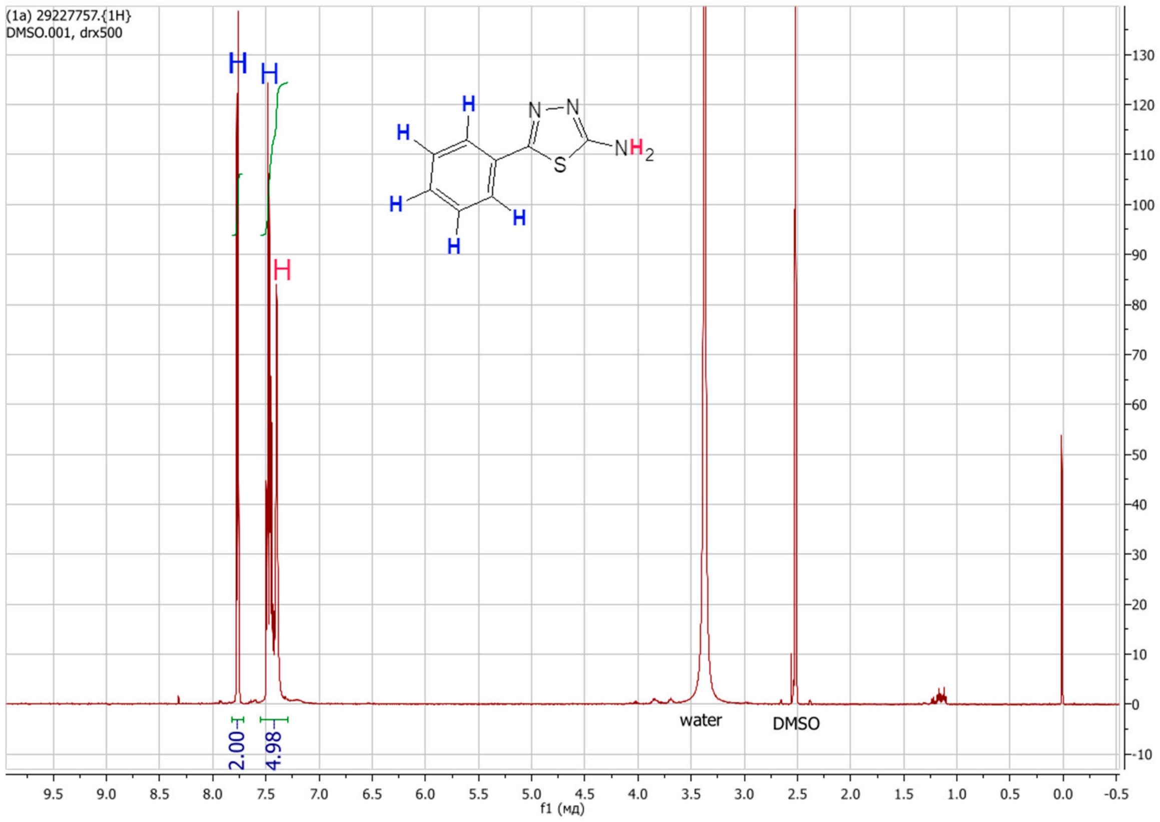

Figure S3. ^{1}H NMR spectrum (DMSO-*d*6) of 5-phenyl-1,3,4-thiadiazol-2-amine (**1a**).

5-(2-Phenylethyl)-1,3,4-thiadiazol-2-amine (1b)

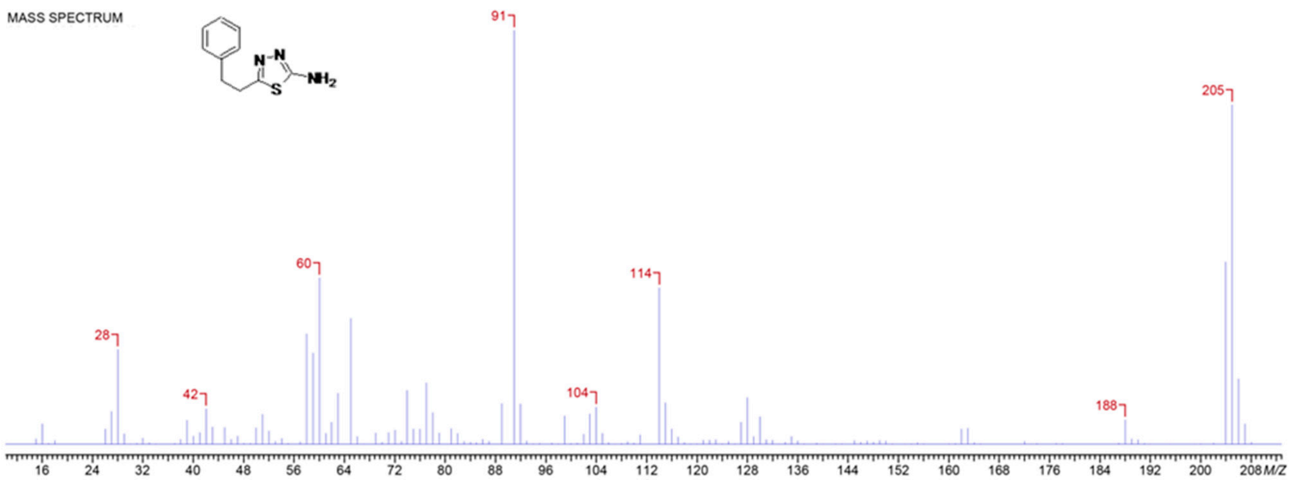

Figure S4. Mass spectrum of 5-(2-phenylethyl)-1,3,4-thiadiazol-2-amine (**1b**).

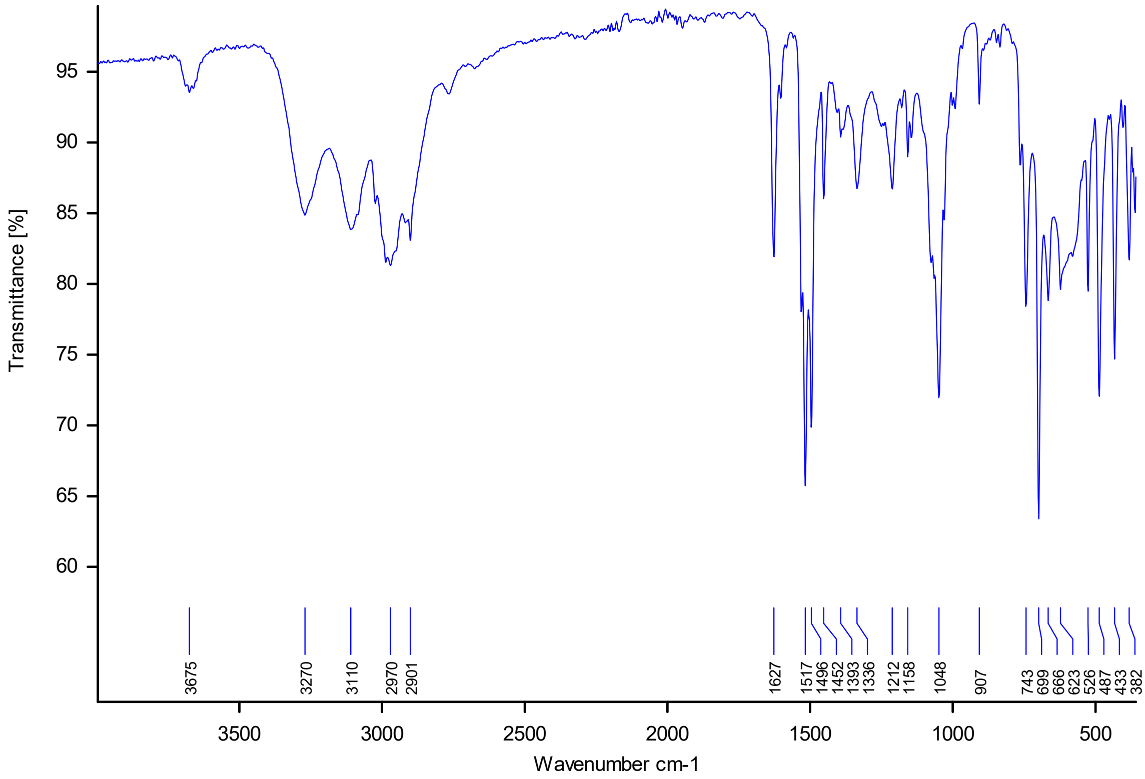

Figure S5. IR spectrum of 5-(2-phenylethyl)-1,3,4-thiadiazol-2-amine (**1b**).

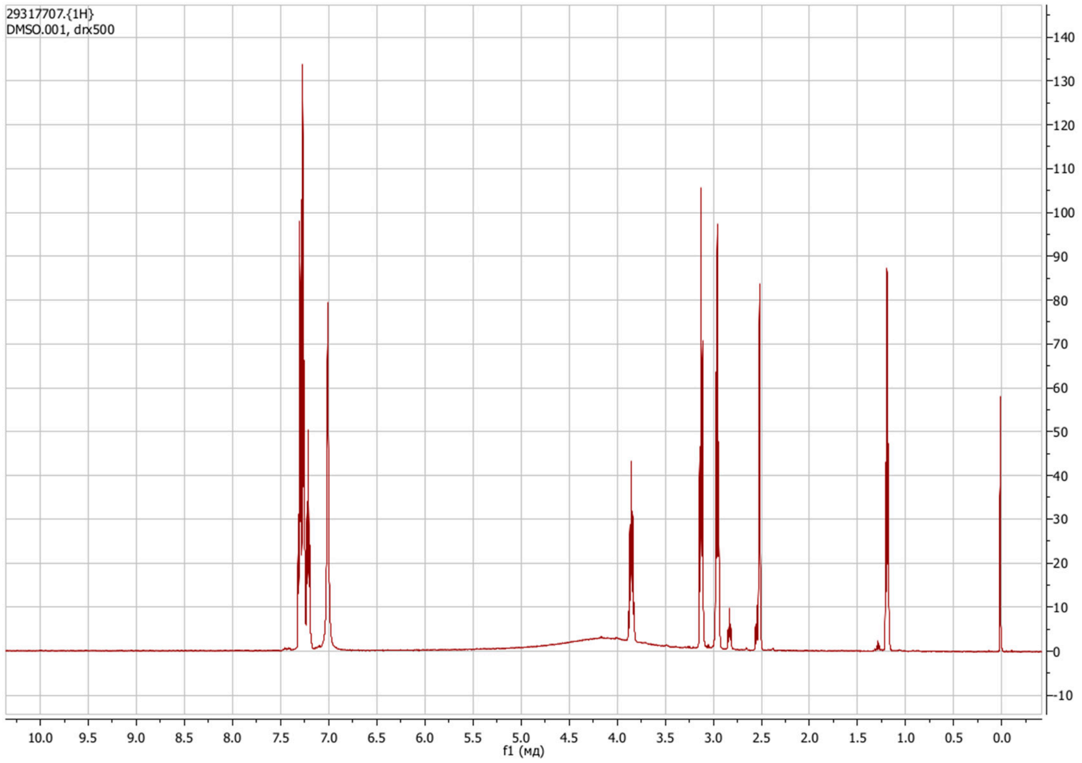

(a)

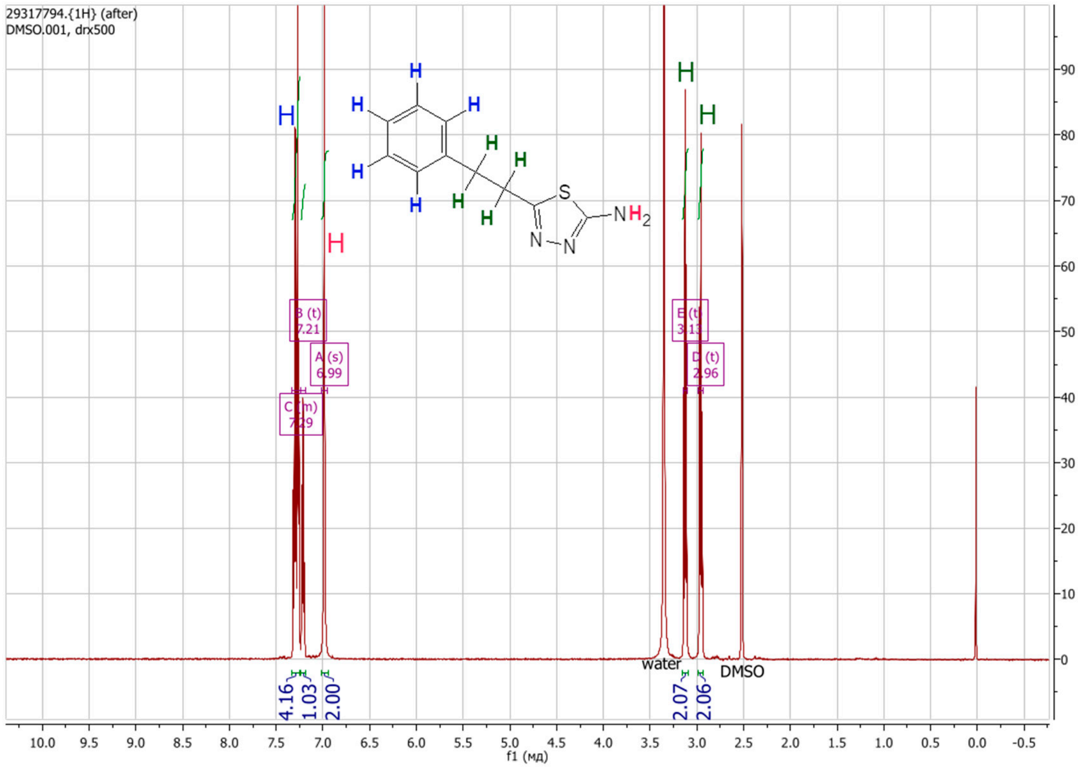

(b)

Figure S6. ^{1}H NMR spectrum (DMSO-*d*$_6$) of 5-(2-phenylethyl)-1,3,4-thiadiazol-2-amine (**1b**) before (a) and after (b) the treatment by hydrochloric acid solution.

5-(Phenoxymethyl)-1,3,4-thiadiazol-2-amine (1c)

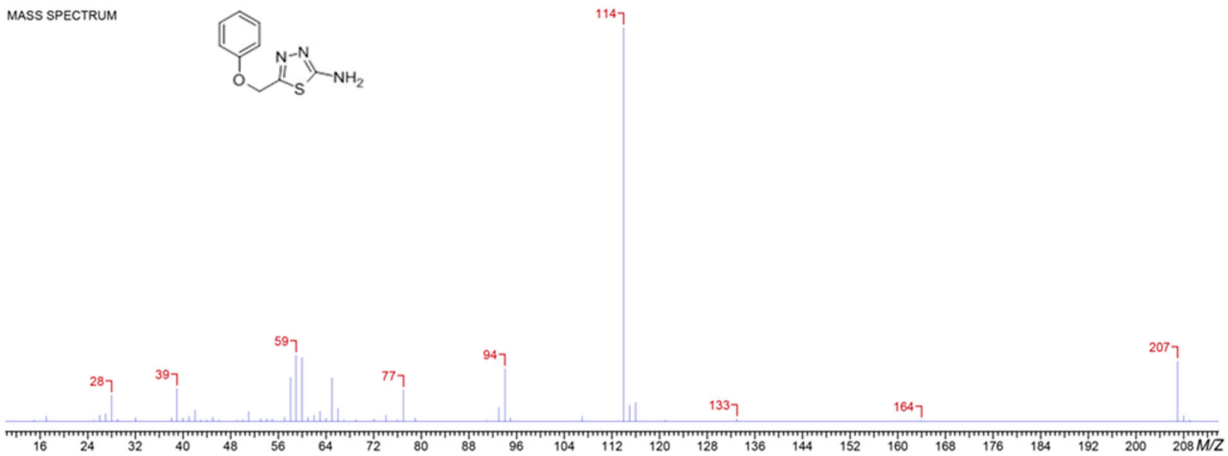

Figure S7. Mass spectrum of 5-(phenoxymethyl)-1,3,4-thiadiazol-2-amine (**1c**).

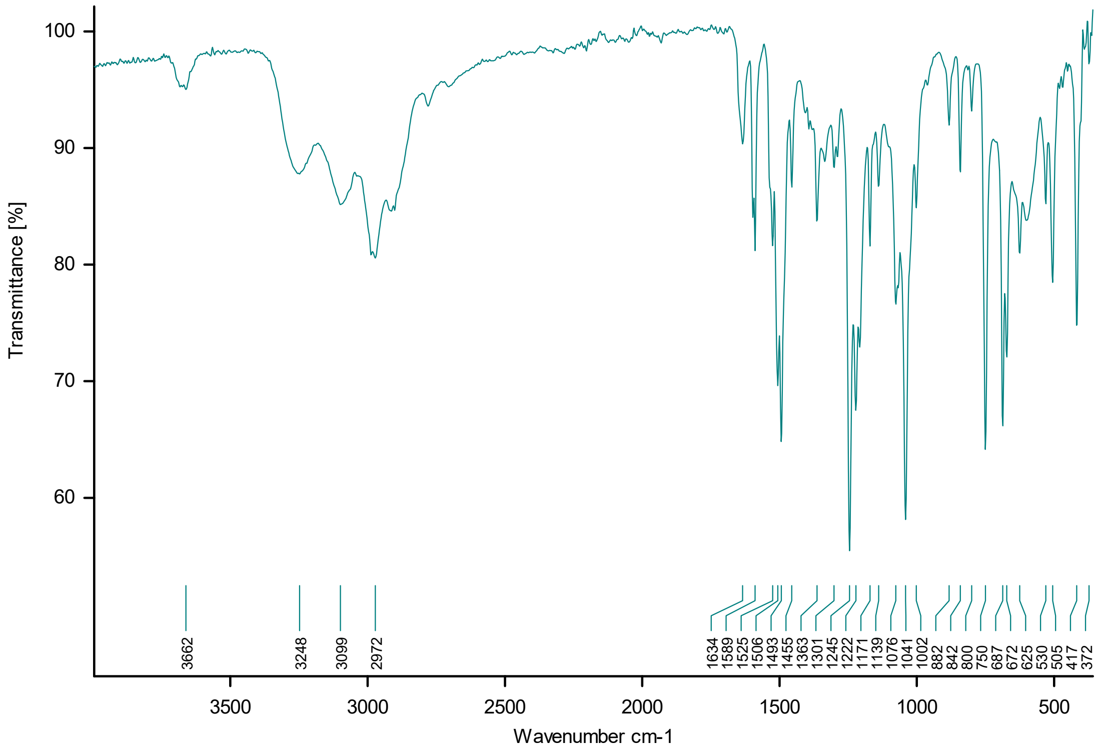

Figure S8. IR spectrum of 5-(phenoxymethyl)-1,3,4-thiadiazol-2-amine (**1c**).

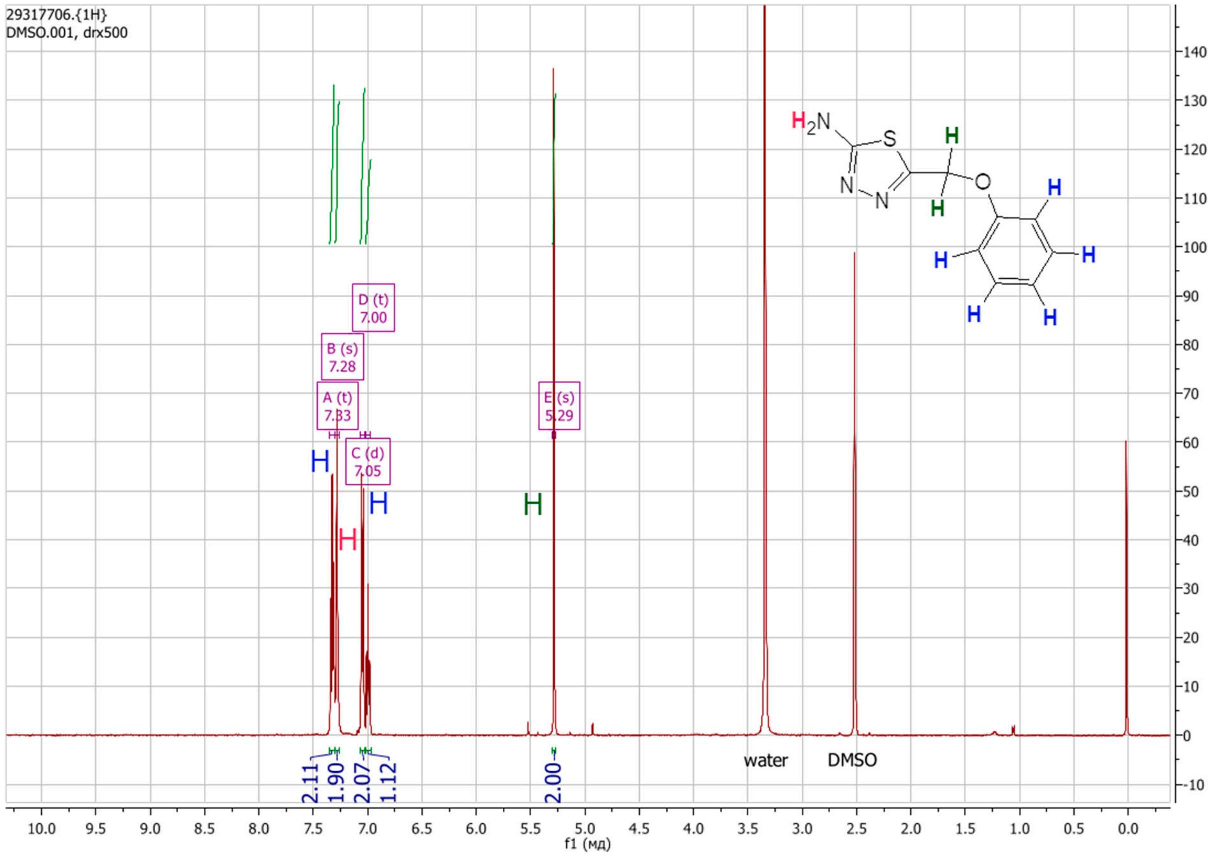

Figure S9. ^{1}H NMR spectrum (DMSO-d_6) of 5-(phenoxymethyl)-1,3,4-thiadiazol-2-amine (**1c**).

5-[(Naphthalen-2-yl)methyl]-1,3,4-thiadiazol-2-amine (1d)

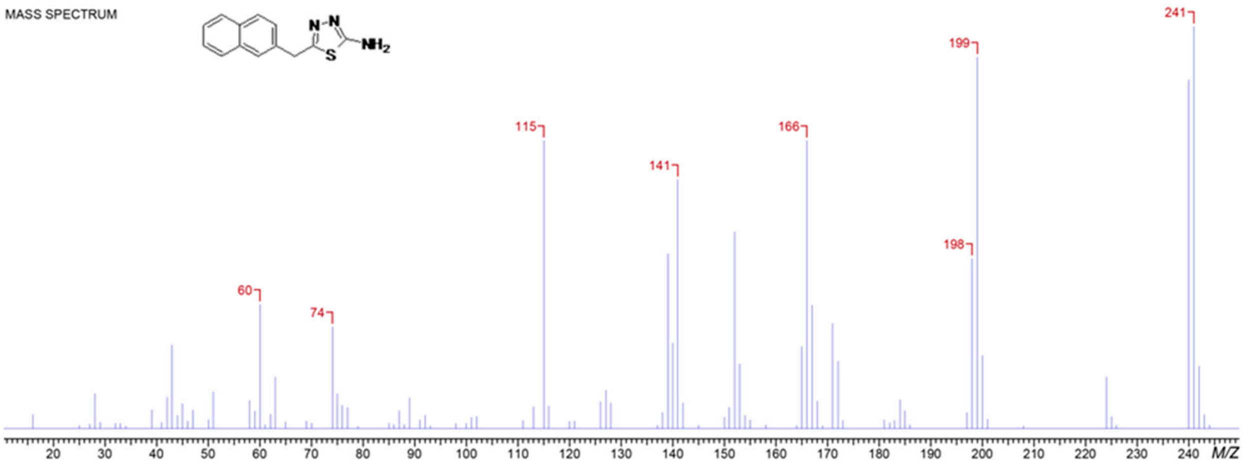

Figure S10. Mass spectrum of 5-[(naphthalen-2-yl)methyl]-1,3,4-thiadiazol-2-amine (**1d**)

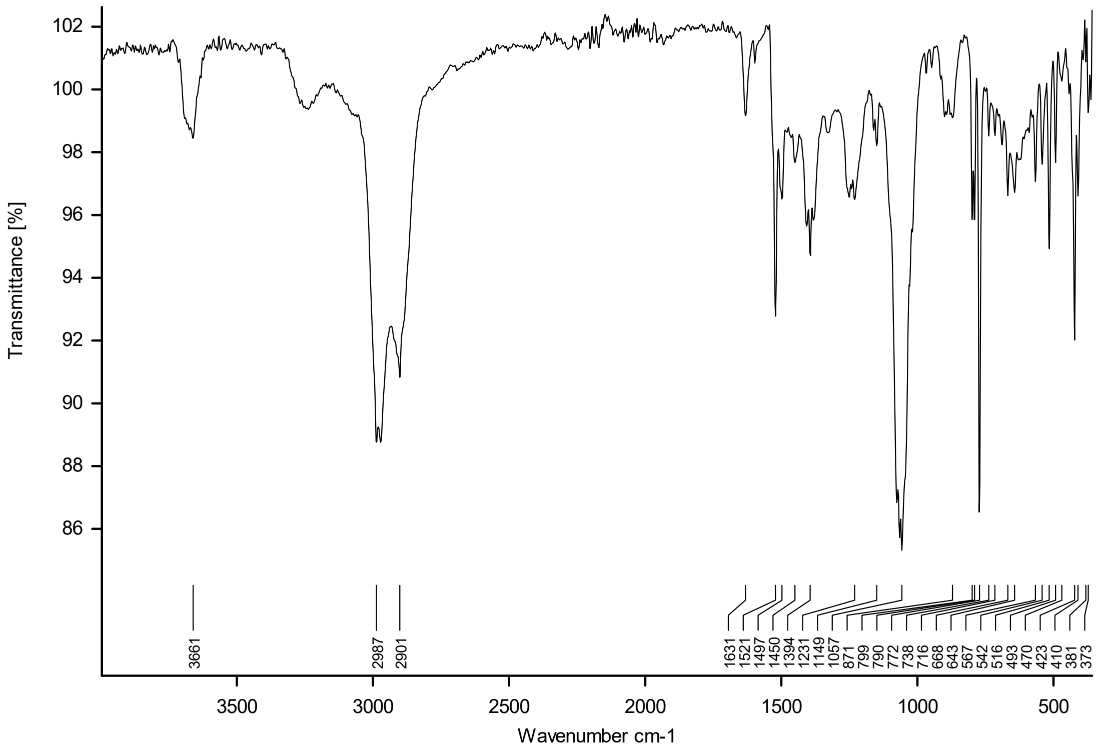

Figure S11. IR spectrum of 5-[(naphthalen-2-yl)methyl]-1,3,4-thiadiazol-2-amine (**1d**)

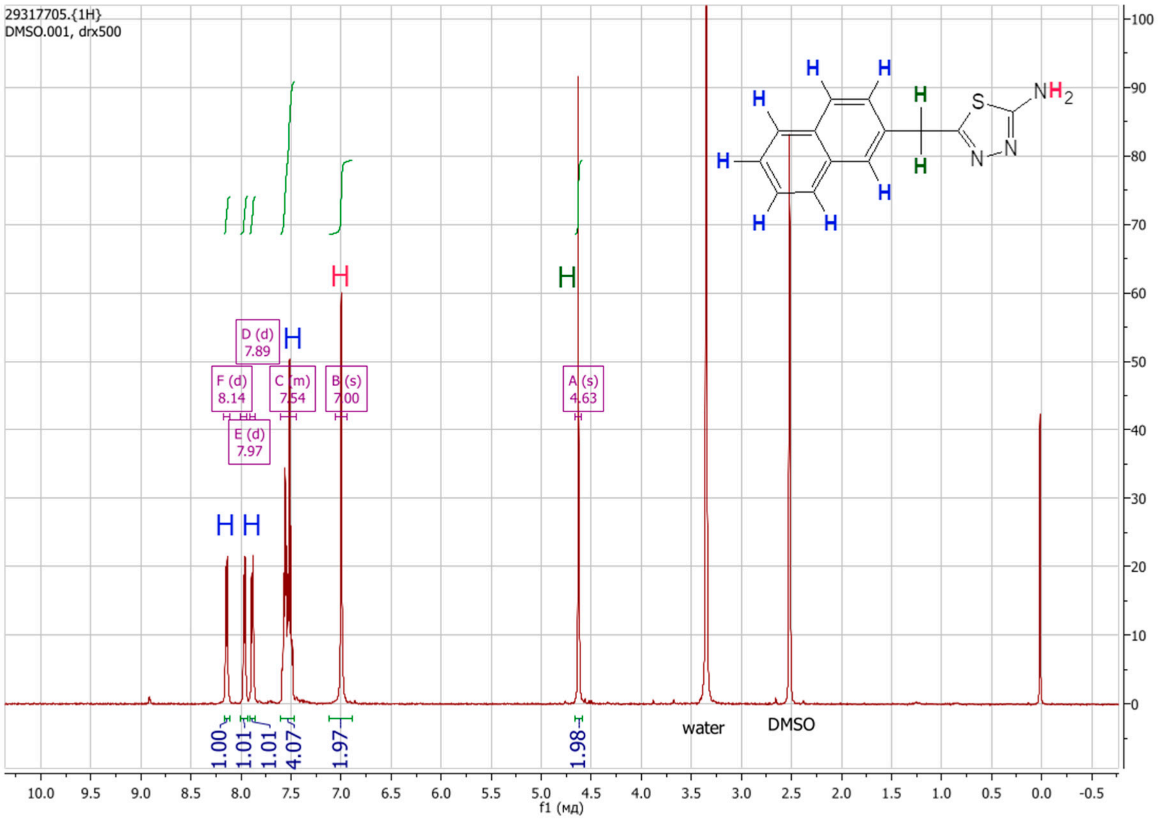

Figure S12. ^{1}H NMR spectrum (DMSO-d_6) of 5-[(naphthalen-2-yl)methyl]-1,3,4-thiadiazol-2-amine (**1d**)

5,5'-(Butane-1,4-diyl)di(1,3,4-thiadiazol-2-amine) (1e)

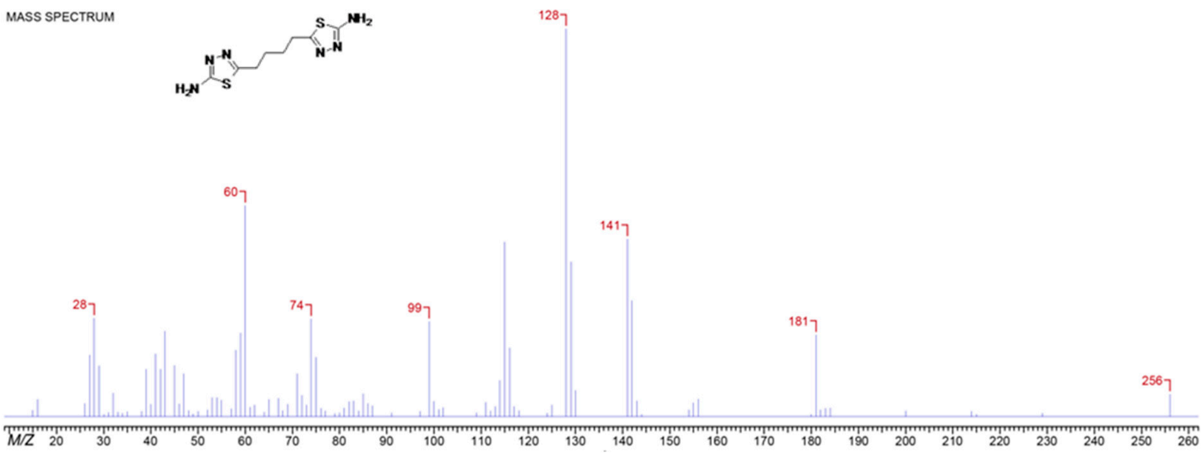

Figure S13. Mass spectrum of 5,5'-(butane-1,4-diyl)di(1,3,4-thiadiazol-2-amine) (**1e**)

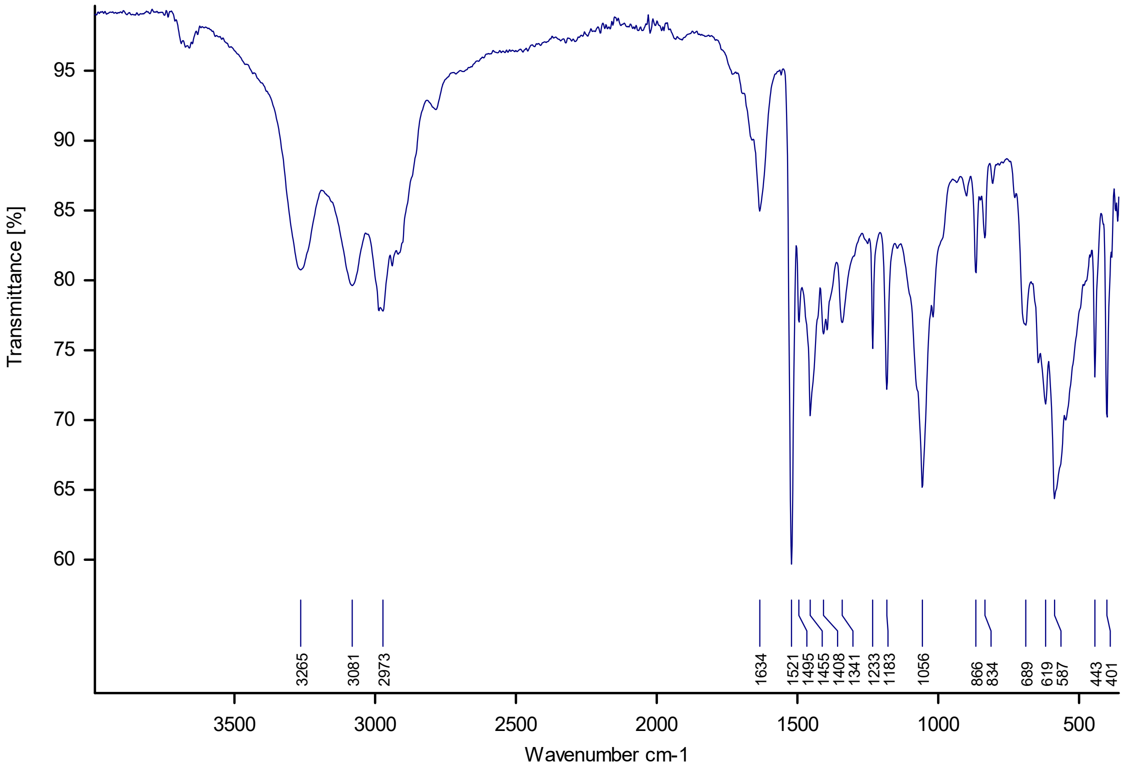

Figure S14. IR spectrum of 5,5'-(butane-1,4-diyl)di(1,3,4-thiadiazol-2-amine) (**1e**)

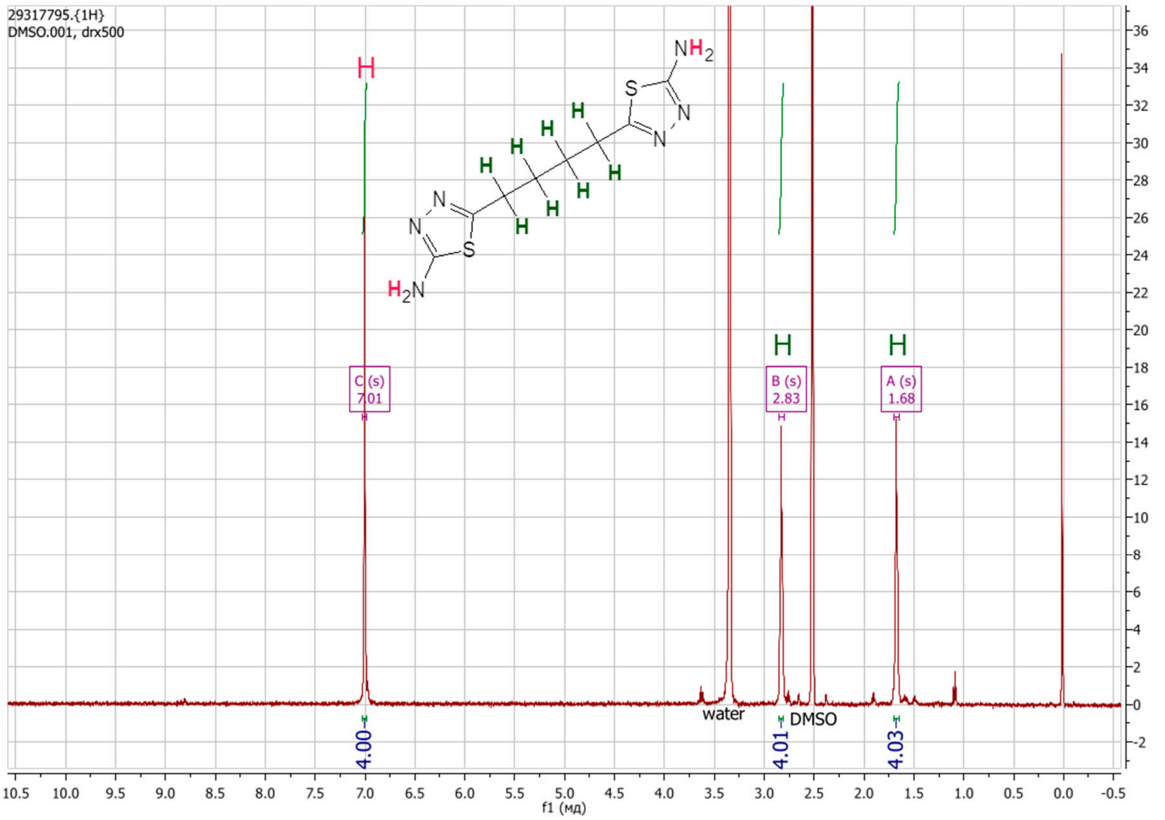

Figure S15. ^{1}H NMR spectrum (DMSO-d_6) of 5,5'-(butane-1,4-diyl)di(1,3,4-thiadiazol-2-amine) (**1e**)

2-Benzoylhydrazine-1-carbothioamide i-1a

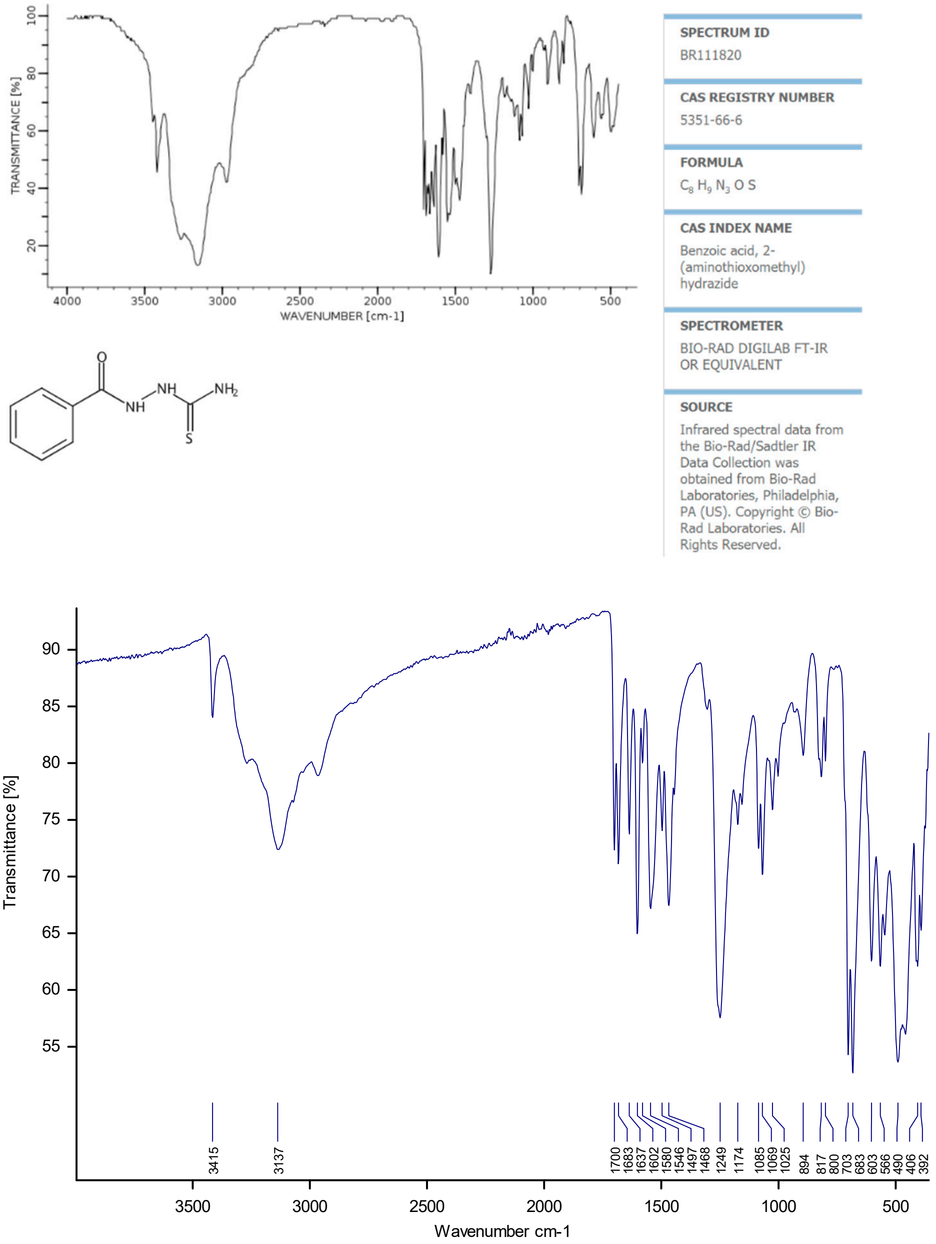

Figure S16. IR spectrum of 2-benzoylhydrazine-1-carbothioamide **(i-1a)** and its comparison with BIO-RAD data base (accessed through the Sci-Finder: https://scifinder.cas.org)

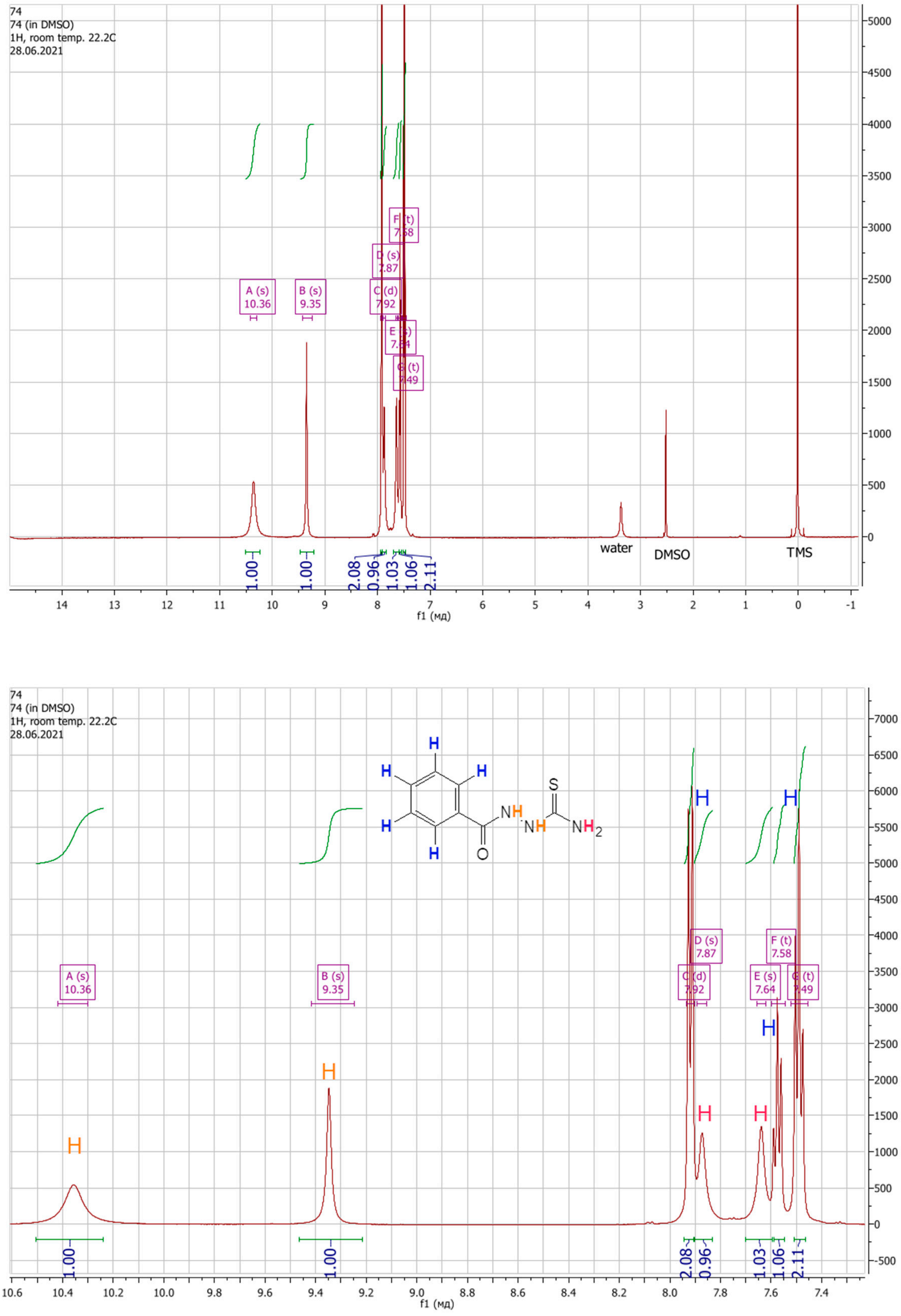

Figure S17. ^{1}H NMR spectrum (DMSO-d_6) of 2-benzoylhydrazine-1-carbothioamide (**i-1a**).
